# Surgical Management of Severe Drug‐Induced Gingival Hypertrophy: A Case Report

**DOI:** 10.1002/ccr3.71128

**Published:** 2025-10-15

**Authors:** John J. Alfarone, Adam Hatala, Dhruv Patel, Sherard Tatum

**Affiliations:** ^1^ Norton College of Medicine SUNY Upstate Medical University Syracuse New York USA; ^2^ Department of Otolaryngology and Communication SUNY Upstate Medical University Syracuse New York USA

**Keywords:** drug‐induced gingival hypertrophy, ear, gingival overgrowth, immunology, nose & throat, oral cavity surgery, otolaryngology

## Abstract

Gingival enlargement is an adverse effect of certain medications, most notably calcium channel blockers, immunosuppressants, and anticonvulsants. We report the case of a 15‐year‐old male with end‐stage renal disease who developed severe drug‐induced gingival enlargement (DIGE) following a deceased donor renal transplant. His immunosuppressive regimen included tacrolimus, prednisone, mycophenolate mofetil, and thymoglobulin, later supplemented with nifedipine for hypertension. Three years after transplant, he developed progressively severe gingival enlargement, leading to impaired mastication, weight loss, and symptoms of sleep‐disordered breathing. Examination revealed marked maxillary and mandibular hyperplasia with pedunculated lesions and enlarged tonsils. Imaging confirmed vascular gingival lesions without bony involvement. The patient underwent operative debulking of the gingival tissue using a Gigli saw and scissors, with adjunctive hemostatic measures, as well as tonsillectomy. Histopathology confirmed fibromatosis with chronic inflammation consistent with DIGE. Postoperatively, the patient demonstrated significant clinical improvement, with weight gain, restoration of oral intake, and satisfactory gingival healing at 3 weeks. This case highlights the complex interplay between immunosuppressants, calcium channel blockers, and oral health in pediatric transplant recipients. Although minor DIGE may regress with medication adjustment and improved oral hygiene, severe cases often necessitate surgical management. Early recognition, interdisciplinary collaboration, and timely surgical intervention are essential to restore function and quality of life in affected patients, particularly those who are immunosuppressed.

## Graphical abstract

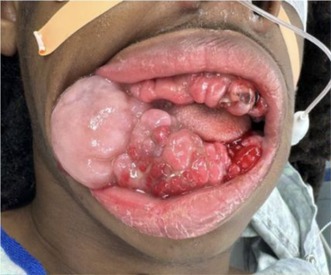

**Summary**

- Drug‐induced gingival enlargement results from the combined effects of medication and local inflammation related to plaque and oral hygiene.
- Accurate diagnosis and comprehensive evaluation are essential before treatment.
- Multidisciplinary care between dentistry, otolaryngology, and nephrology supports timely recognition, coordinated management, and appropriate intervention, including surgery in severe pediatric cases.

## 1 Introduction

Gingival enlargement, also known as gingival hypertrophy, refers to exaggerated growth of the periodontal soft tissues [1]. This is often an adverse reaction to certain medications, including calcium channel blockers (CCBs), immunosuppressants, and anticonvulsants [1, 2]. Severity may be worsened by dental plaque, which is often linked to poor oral hygiene [2].

We report a case of a 15‐year‐old male with a history of end‐stage renal disease (ESRD) who developed severe gingival enlargement secondary to prolonged therapy with immunosuppressants and calcium channel blockers following a deceased donor renal transplant (DDRT). This case highlights the surgical management of severe drug‐induced gingival enlargement (DIGE).

## 2 Case History/Examination

A 15‐year‐old male with ESRD secondary to renal dysplasia and posterior urethral valves underwent DDRT at age 12. He had been on hemodialysis for 2 years prior to transplantation.

His baseline dental history was notable for longstanding poor dentition, documented as early as 2016 (5 years before DDRT). In the months preceding DDRT, he required formal dental clearance, which included extensive plaque removal and professional cleaning.

Postoperatively, his regimen included 3 mg tacrolimus twice a day, 20 mg prednisone daily, mycophenolate mofetil 3 tablets (540 mg) orally every morning, two tablets (360 mg) every evening, and 6 mg/kg of thymoglobulin IV for four doses. The transplant team set a target systolic blood pressure (SBP) of 120–140 mmHg, which was initially achieved. However, after tacrolimus initiation, his SBP consistently exceeded 140 mmHg, and amlodipine 2.5 mg BID was started.

Fifteen months after DDRT, amlodipine was stopped, and nifedipine 30 mg was started following a kidney biopsy that showed arteriolar thickening suggestive of poorly controlled hypertension.

Two years after DDRT, (9 months after initiation of nifedipine), the patient developed mild gingival enlargement, which did not impact his quality of life and remained stable for approximately 1 year.

Three years after DDRT (1 year after the onset of mild gingival enlargement), the condition began to impair eating. The family reported missed dental appointments and oral hygiene becoming a challenge at home. The patient was admitted due to a 10‐pound weight loss from an inability to chew or eat solid food and showed symptoms of sleep‐disordered breathing.

**FIGURE 1:**
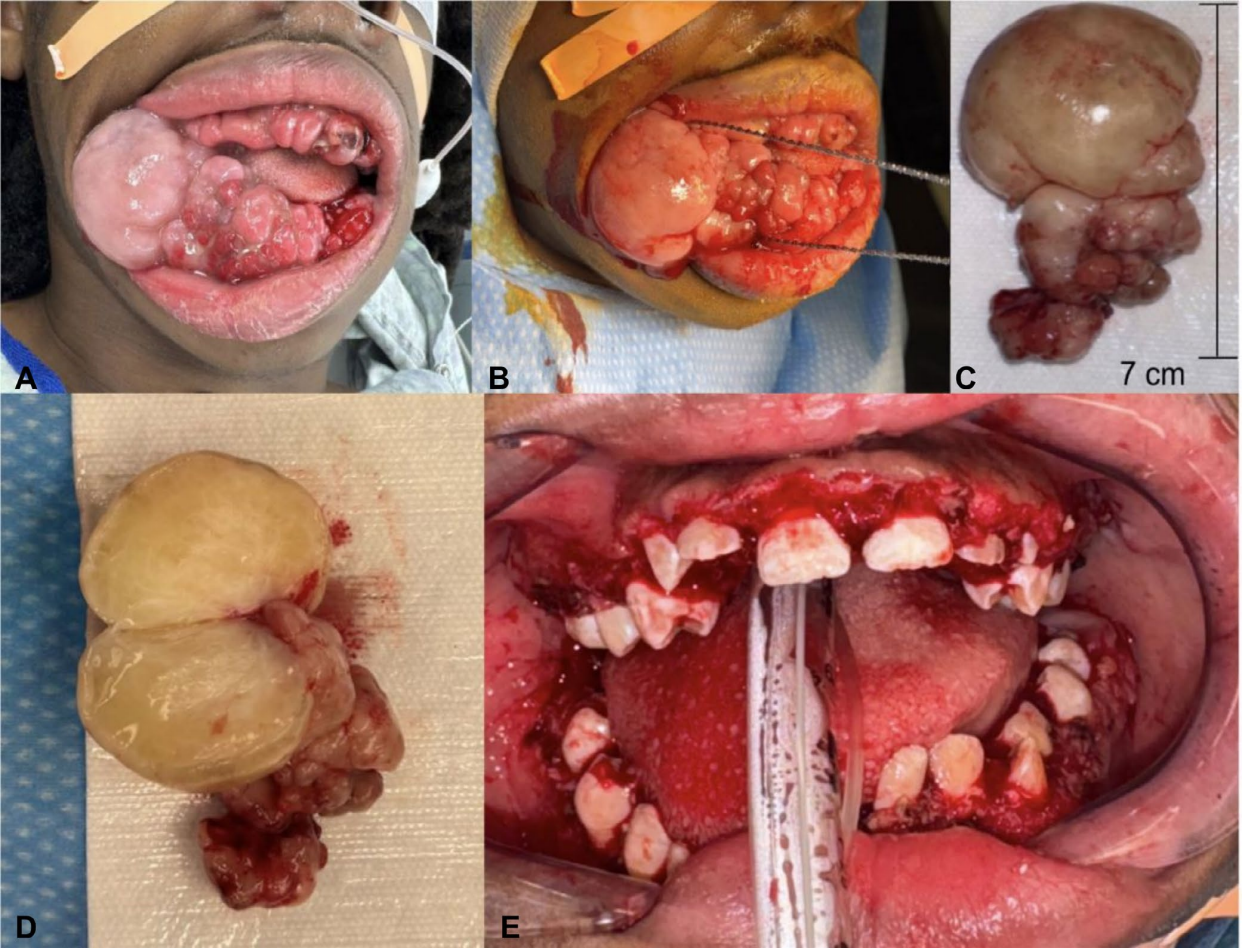
(A) Hyperplastic changes of the maxillary and mandibular gingiva with pedunculated lesion along the right mandible. (B) Intra‐operative view showing Gigli saw around the large pedunculated lesion. (C) Resected pedunculated lesion measuring 7 cm in greatest dimension. (D) Transected right‐sided lesion after Gigli saw excision. (E) Immediate postoperative view showing successful debulking and restoration of the gingival contours, with improved visualization of the dentition.

On exam, enlargement was noted along the maxillary and mandibular gingiva, with pedunculated portions along the right mandible and enlarged tonsils (Figure 1A).

## 3 Differential Diagnosis, Investigations, and Treatment

Prior to the initiation of nifedipine, routine intraoral examinations documented poor dentition and plaque accumulation but no gingival enlargement. The gingival tissues appeared within normal limits without hyperplasia or pedunculated growths. This baseline status supports a causal relationship between nifedipine initiation and the subsequent development of gingival enlargement.

Thirty‐six months after DDRT, and approximately 21 months after initiation of nifedipine, the medication was discontinued per pediatric nephrology, and Otolaryngology–Head and Neck Surgery was consulted. Due to the nutritional challenges, the patient was considered for operative debulking of the oral cavity lesions. To better evaluate the lesions, magnetic resonance imaging (MRI) with contrast was obtained and revealed large vasculature throughout the lesions, all emanating from the gingiva. Computed tomography (CT) without contrast was also obtained to investigate bony involvement; contrast was deferred due to renal impairment. The patient was typed and cross‐matched in preparation for significant blood loss. Given the enlarged tonsils, sleep‐disordered breathing, and immunosuppression, tonsillectomy was also planned to reduce obstruction and rule out underlying lymphoproliferative disorders. Leukemic gingival infiltration and hereditary gingival fibromatosis were considered as differential diagnoses, but with lower clinical suspicion as the patient did not have any previous lab work revealing anemias, leukocytosis, or pancytopenias, nor a family history of gingival enlargement.

In the operating room, the patient was initially fiberoptically nasally intubated to allow for maximal intraoral space. Preoperatively, we planned to minimize electrocautery to limit disruption of the submucosa and periosteum and enhance gingival healing. Because the condition is typically limited to attached gingiva and spares vestibular mucosa, the large lesion was thought to be pedunculated with a base limited to the gingiva. A Gigli saw was passed around the stalk of the large pedunculated lesion, which was excised (Figure 1B–D). Excess gingiva was excised from right to left along the buccal and lingual mandible using straight and curved Mayo scissors in a stepwise fashion. Bleeding was managed with direct pressure, Floseal (BAXTER, Deerfield, IL), QuikClot (Z‐Medica, Wallingford, CT), and minimal Bovie electrocautery. Most of the large supplying vessels came from the interdental papilla. Dentition was re‐exposed using a #15 blade and iris scissors to incise the gingival overgrowth over the occlusal surfaces. The lingual mandibular gingiva was also debulked, and the same technique was applied to the maxilla. Mandibular and maxillary dentition were exposed and showed significant malocclusion and plaque buildup. The dental team assisted with plaque removal under anesthesia. The patient was then transitioned to orotracheal intubation, and a standard tonsillectomy was performed. Histopathological examination demonstrated fibromatosis with dense collagenous connective tissue, moderate lymphoplasmacytic chronic inflammation, and reactive hyperplastic squamous mucosa (findings consistent with drug‐induced gingival enlargement). Tonsillar specimens showed reactive lymphoid tissue with mild acute inflammation, with no evidence of lymphoproliferative disease.

## 4 Conclusion and Results (Outcome and Follow‐Up)

Postoperatively (Figure 1E), the patient was placed on a soft, nonchew diet, began a 10‐day course of amoxicillin‐clavulanate, and was instructed to use saline mouth rinses.

**FIGURE 2:**
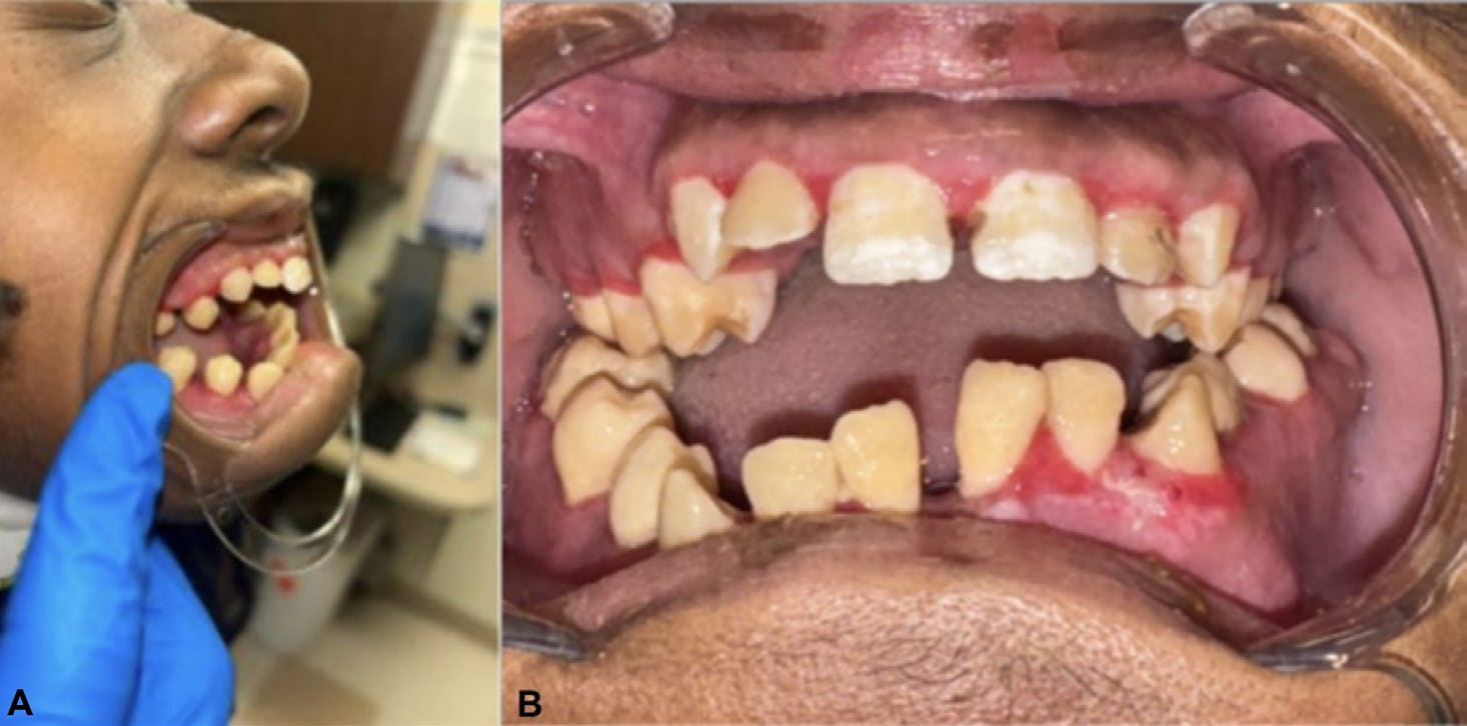
Three‐week postoperative follow‐up showing healed gingiva and improved dentition exposure. (A) Lateral view. (B) Frontal view.

At 3‐week follow‐up, the gingiva was healing well, eating habits had improved, and he regained 29 pounds (Figure 2A,B).

At 2‐month follow‐up, he continued to do well with no evidence of gingival regrowth. He was eating normally, had no systemic symptoms such as fevers or chills, and had recently undergone a routine dental cleaning 2 weeks earlier.

At 5‐month follow‐up, he again demonstrated no signs of recurrent gingival enlargement or abnormal tissue growth. He reported brushing regularly, though not flossing, and was planning for another dental cleaning in the near future.

He continues to follow up with dentistry every 4 months for ongoing surveillance and plaque control.

He has also remained under regular follow‐up with pediatric nephrology, with no recurrence of hypertension requiring calcium channel blocker therapy.

**FIGURE 3:**
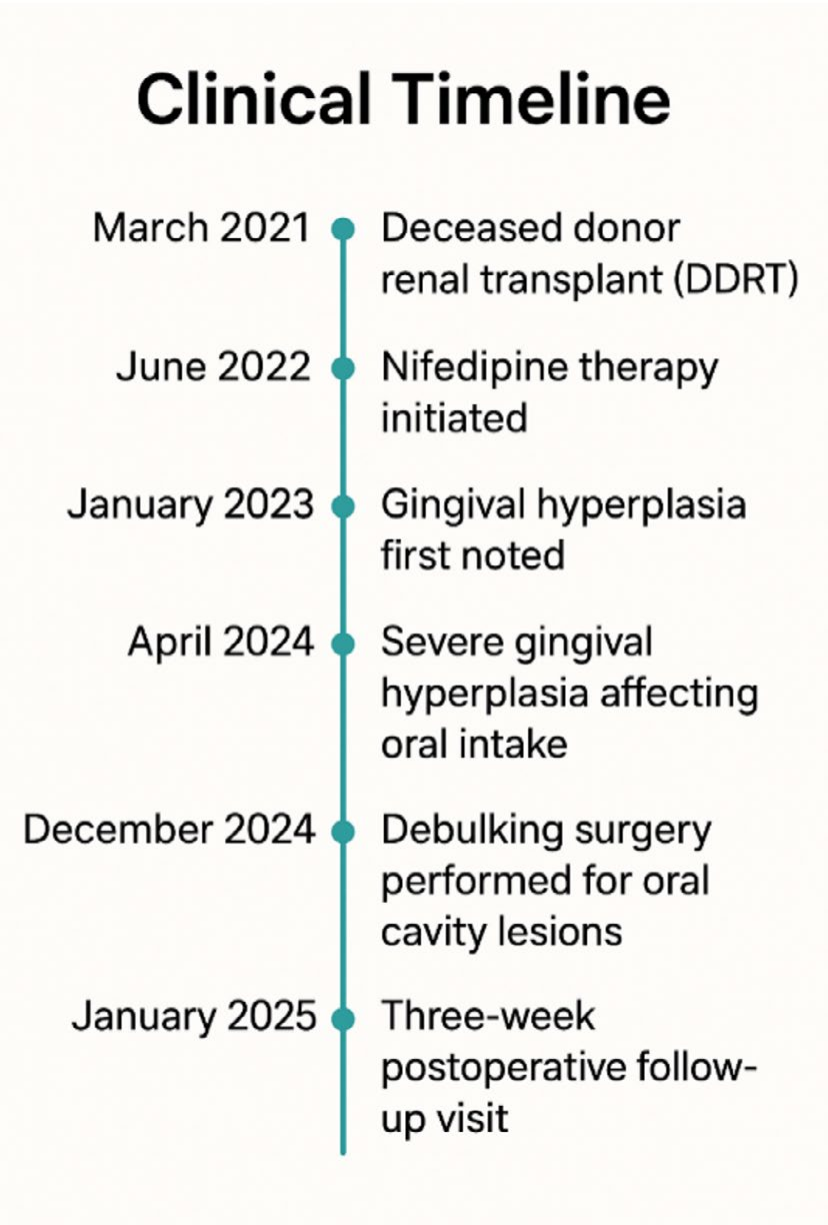
Timeline of key clinical events from deceased donor renal transplant (DDRT) to postoperative follow‐up visit.

A summary of the patient's clinical course, from initial presentation to surgical intervention and initial follow‐up visit, is outlined below in Figure 3.

## 5 Discussion

Gingival enlargement has multiple etiologies, including inflammatory, infiltrative, hereditary, and drug‐induced [3]. DIGE is an adverse effect of several medications, including immunosuppressants such as cyclosporine and tacrolimus, calcium channel blockers such as nifedipine and amlodipine, and anticonvulsants such as phenytoin [2, 4]. The mechanism is complex and not fully understood, but it is thought to involve the inhibition of sodium and calcium influx, leading to decreased folate uptake [2]. Folate plays a key role in DNA synthesis, and its deficiency first affects tissues with high turnover, such as epithelium and bone marrow [2]. Folate is also needed to activate collagenase, an enzyme that cleaves collagen [2]. In folate deficiency, inactive collagenase fails to degrade excess gingival connective tissue, resulting in enlargement [2].

Tacrolimus, a calcineurin inhibitor, is commonly used to prevent organ rejection following transplantation [4]. Compared to cyclosporine, tacrolimus tends to have a more favorable side effect profile, including a lower incidence of hypertension and a lesser chance of causing gingival enlargement [4]. In renal transplant cohorts, gingival overgrowth has been observed in roughly a third of cyclosporine users, while no cases were found among tacrolimus users in one clinical study [5]. Rare case reports describe tacrolimus‐associated enlargement, which underscores the possibility but not probability [4]. Given this evidence and the clinical chronology in our case, tacrolimus is an unlikely principal cause here.

Minor cases of drug‐induced gingival enlargement may be partially reversible if the causative medication is discontinued [3]. However, not all patients will respond, particularly when lesions are longstanding or severe [6]. Current clinical guidance emphasizes that the first line of management should focus on meticulous plaque control and, when medically feasible, modification or discontinuation of the implicated drug, with surgical intervention reserved for persistent or severe cases [6]. More advanced cases that compromise function or quality of life may require gingivectomy to debulk the lesions, as demonstrated in our patient [2, 3]. Pundir et al. noted that a conservative approach consisting of regular professional prophylaxis improved plaque control and reduced the need for surgical management in patients with DIGE [7].

In our case, amlodipine was used early after transplant without clinical evidence of gingival enlargement. At 15 months, amlodipine was replaced with nifedipine. Gingival enlargement was first documented 9 months later and progressed over the following year. Population data support a stronger association between nifedipine and gingival overgrowth than with amlodipine. A community study reported overgrowth in about one in five nifedipine users versus about one in 30 amlodipine users [8]. Another series identified a prevalence near 3% with amlodipine, consistent with a lower risk profile [9]. The absence of enlargement on amlodipine, followed by onset and progression after nifedipine initiation, reinforces nifedipine as the primary driver in this patient.

In this case, drug substitution was not pursued earlier because nifedipine was considered essential by the pediatric nephrology team for the management of post‐transplant hypertension. Preserving adequate blood pressure control was prioritized to support renal graft health, and the early gingival changes were mild and nondisabling. As a result, conservative dental follow‐up was preferred initially. Intervention was delayed until 2 years after onset, when the enlargement progressed to impair mastication, cause weight loss, and contribute to sleep‐disordered breathing, prompting the discontinuation of nifedipine and surgical debulking.

## Author Contributions

**John J. Alfarone:** conceptualization, validation, visualization, writing – original draft, writing – review and editing. **Adam Hatala:** conceptualization, project administration, supervision, validation, writing – original draft, writing – review and editing. **Dhruv Patel:** conceptualization, project administration, supervision, validation, writing – original draft, writing – review and editing. **Sherard Tatum:** project administration, supervision, writing – review and editing.

## Disclosure

The authors have nothing to report.

## Ethics Statement

Our institution does not require ethical approval for reporting individual cases or case series.

## Consent

Informed written consent was obtained from the patient for publication of this case report.

## Conflicts of Interest

The authors declare no conflicts of interest.

## Data Availability

The data that support the findings of this study are available from the corresponding author upon reasonable request.
